# Editorial: Toward a better understanding and application of benefit sharing in genomic and global health research

**DOI:** 10.3389/fgene.2023.1291181

**Published:** 2023-09-27

**Authors:** Aminu Yakubu, Ann M. Mc Cartney, Dominique Sprumont

**Affiliations:** ^1^ Center for Bioethics and Research, Ibadan, Nigeria; ^2^ 54gene Inc., Washington, DC, United States; ^3^ Genomics Institute, University of California, Santa Cruz, Santa Cruz, United States; ^4^ Institute of Health Law, University of Neuchâtel, Neuchâtel, Switzerland

**Keywords:** benefit sharing, genetics research, indigenou peoples, digital sequence information (DSI), biobanking research, federated data access

Philosophically grounded in the principle of beneficence, discourse on the issue of benefits and benefit sharing in biomedical research has been ongoing for decades but has received additional impetus with the recent growth of genetics and genomics research. Over the past 2 decades an international consensus has been built highlighting the need for both compensatory and distributive justice through benefit sharing with research participants and their communities (Hugo ethics committee, 2000). Although initially applied in regard to therapeutic benefit sharing from from clinical trials, this consensus has expanded to encompass benefits across all biomedical research including: genetic and non-genetic human subject research (White, 2007; Dauda and Dierickx, 2017; Bedeker et al., 2022), human pathogen research (Rourke, 2017), research involving access and use of biodiversity as well research involving Indigenous Knowledges (Heinrich et al., 2020; Tone-Pah-Hote and Redvers, 2022). It has been noted that existing national and international guidelines have failed to offer practical and transparent guidance to support biomedical researchers to share the benefits from their research (Sudoi et al., 2021). Additionally, although there are few documented cases of successful benefit sharing in practice, cases of poor benefit sharing implementation are more common with the most recent and visible case being theSARS-CoV 2 vaccine development and the distribution of benefits (Hassan et al., 2021; Sekalala et al., 2021). The Research Topic of articles presented in this issue aimed to pool together additional literature from a wide pool of researchers to add to the body of evidence on the issue of benefit sharing with the hope of addressing some of the gaps in practice and regulation.

From an African communitarian perspective, Ewuoso et al. proposed a set of normative principles that should inform the ethical foundation of benefit sharing. In the article, they argue that the principles of humanness, friendliness and partiality, when applied to genetics research, could facilitate “thinking critically about why benefit sharing, what may count as benefits within the context of human research in Africa and the limits of the obligation of benefit sharing”. These norms add to others that have been proposed in support of benefit sharing including solidarity and justice by the HUGO ethics committee (Hugo ethics committee, 2000), for example. Although the manuscript was drafted within an African context, the broad principles argued for could be useful and inform benefit sharing practices beyond African communities.

From the human biomedical research perspective, Alvarellos et al. looked at the need to democratise clinical genomics research to improve clinical practice and research as an important benefit sharing approach. They discussed how federated data platforms could be applied to facilitate ethical sharing of clinical data across institutions and geographies. Practical examples on the adoption of federated platforms are mostly limited to high income countries and the Global North, except the Common Infrastructure for National Cohorts in Europe, Canada, and Africa (CINECA) project, which is working to include data from Europe, Canada and Africa, with South Africa being the only African country so far included in this initiative. However, there are additional outstanding concerns that would require consideration if federated platforms were to adopted as means to equitably democratise genomic data among global populations, including the limited inclusion of African and other diverse population data in genomics research, challenges in access to and affordability of technologies to access federated platforms and the lingering concern about trust (Thorogood et al., 2021) are.

Mwaka et al. shared perspectives from researchers and research ethics committees in Uganda on benefit sharing. Respondents were concerned about challenges to equitable sharing of benefits from international collaborative genomics and biobanking research with researchers, communities and participants. They believed researchers, communities and participants should benefit directly from genetic and biobanking research (GBR) - there is however, lack of clarity in Ugandan regulations on how this can be achieved. This lack of clear regulations on GBR is reported to be a general concern in African countries as echoed in reviews, including a recent review by Ali and his colleagues (Mulder et al., 2017; Ali et al., 2021). As developments in this regard emerge, African regulators have been cautioned to consider regulations that balance the need to provide adequate protections for researchers and participants from Africa, while facilitating international research collaborations (de Vries et al., 2017; Kabata and Thaldar, 2023).

The role of benefit sharing in promoting the use of genetic information in medicines development was discussed by Matsuyama et al. Within the contexts of population genomics, pharmacogenomics, and barriers to global health attendant to patent restrictions on products of genomics research, the authors argued for the adoption of fair benefit sharing principles as a solution. In the manuscript they considered research information and products developed from research on “public goods” and addressed how to promote benefit sharing considerations that are more fair to stakeholders in the Global North and South than currently unfair practices including recent experiences in access to COVID-19 vaccines.

For non-human subjects genetics research provisions of benefit sharing from research involving the access and use of biodiversity genetics resources falls under the auspices of Convention on Biological Diversity’s (“CBD”) Nagoya Protocol on Access and Benefit Sharing, this includes Indigenous genetics resources and Traditional Knowledge. Although the Protocol offers more clear expectations for biodiversity genetics research, Golan et al. problematized its provisions arguing that it does not yet clearly address the issue of benefit sharing when for the access and use of the sequencing information associated with biodiversity genetics resources, which is more commonly referred to as Digital Sequence Information (DSI). They propose the mainstreaming of the use of a digital labelling system, such as that offered by the Traditional Knowledge and Biocultural Labels and Notices, to provide permanent links between DSI and accurate provenance information. However there are others contributing to the discourse on the fairness and equitable nature of the Nagoya Protocol. For instance, a study from six Latin countries reported broader problems to Nagoya Protocol implementation including weak institutional infrastructures to implement national policies, and limited knowledge about the Protocol and the CBD within countries (Heinrich et al., 2020). While the proposal by Golan and colleagues is laudable, these broader challenges to the ethical use of Indigenous genetics resources need to be addressed for a more lasting benefit sharing regime. In a related article, Carroll et al. outline how the four CARE (Collective Benefit, Authority to Control, Responsibility, Ethics) Principles for Indigenous data governance can be applied to benefit sharing in Indigenous genomics research. Benefits that could apply to research involving Indigenous populations applying the CARE principles range from compensation in general (not restricted monetary payments to research participants), payment of royalties, commercialization agreements (and provisions for restrictions), return of results, co-authorship and ethical oversight to ensure fair risk benefit balance (Carroll et al., 2020). Arguably, this is a practical framework that could codify benefit sharing in indigenous research and improve involvement of Indigenous people in governance of research (Jennings et al., 2023; Mc Cartney et al., 2023).

Collectively, the articles in this Research Topic have succeeded in providing additional literature on the issue of benefit sharing in biomedical research that could contribute towards more internationally acceptable practical frameworks. One such framework has already been proposed by Bedeker and colleagues that looks at benefit sharing at two strategic levels - 1) stakeholders that may receive benefits with examples; and 2) description of different categories of benefits that sponsors and researchers could consider in the design of calls for research grants, research proposals and/or benefit sharing negotiations (Bedeker et al., 2022). The extent to which this framework solves the problem of addressing the issue of benefit sharing in practice is left to be seen. However, what this Research Topic clearly demonstrates is that the issue of benefit sharing in biomedical research remains an important concern in ensuring equitable and fair research within countries and across international boundaries. More research is needed on who, how and when benefits ought to be shared and how benefit sharing ought to be regulated for human genetics research, specifically whether non-legally binding regulations are sufficient or whether an internationally binding treaty or protocol on benefit sharing should be considered. In addition, more clear guidelines that can help with more sustainable implementation of benefit sharing in biomedical research are needed that prioritise the principles of fairness, equity, and solidarity.

## References

[B1] Ali J. Cohn B. Mwaka E. Bollinger J. M. Kwagala B. Barugahare J. (2021). A scoping review of genetics and genomics research ethics policies and guidelines for Africa. BMC Med. Ethics 22, 39. 10.1186/s12910-021-00611-9 33810790 PMC8017870

[B2] Bedeker A. Nichols M. Allie T. Tamuhla T. van Heusden P. Olorunsogbon O. (2022). A framework for the promotion of ethical benefit sharing in health research. BMJ Glob. Health 7, e008096. 10.1136/bmjgh-2021-008096 PMC8845198 35144922

[B3] Carroll S. R. Garba I. Figueroa-Rodríguez O. L. Holbrook J. Lovett R. Materechera S. (2020). The CARE principles for indigenous data governance. CODATA 19, 43. 10.5334/dsj-2020-043

[B4] Dauda B. Dierickx K. (2017). Viewing benefit sharing in global health research through the lens of Aristotelian justice. J. Med. Ethics 43, 417–421. 10.1136/medethics-2015-102858 28550158

[B5] de Vries J. Munung S. N. Matimba A. McCurdy S. Ouwe Missi Oukem-Boyer O. Staunton C. (2017). Regulation of genomic and biobanking research in Africa: A content analysis of ethics guidelines, policies and procedures from 22 african countries. BMC Med. Ethics 18, 8. 10.1186/s12910-016-0165-6 28153006 PMC5289015

[B6] Hassan F. Yamey G. Abbasi K. (2021). Profiteering from vaccine inequity: A crime against humanity? BMJ 374, n2027. 10.1136/bmj.n2027 34400410

[B7] Heinrich M. Scotti F. Andrade-Cetto A. Berger-Gonzalez M. Echeverría J. Friso F. (2020). Access and benefit sharing under the Nagoya protocol-quo vadis? Six Latin American case studies assessing opportunities and risk. Front. Pharmacol. 11, 765. 10.3389/fphar.2020.00765 32581783 PMC7294742

[B8] Hugo ethics committee (2000). Hugo ethics committee statement on benefit sharing: april 9, 2000. Clin. Genet. 58, 364–366. 10.1034/j.1399-0004.2000.580505.x 11140835

[B9] Jennings L. Anderson T. Martinez A. Sterling R. Chavez D. D. Garba I. (2023). Applying the “CARE principles for indigenous data governance” to ecology and biodiversity research. Nat. Ecol. Evol. 10.1038/s41559-023-02161-2 37558804

[B10] Kabata F. Thaldar D. (2023). Regulating human genomic research in Africa: why a human rights approach is a more promising conceptual framework than genomic sovereignty. Front. Genet. 14, 1208606. 10.3389/fgene.2023.1208606 37456664 PMC10347388

[B11] Mc Cartney A. M. Head M. A. Tsosie K. S. Sterner B. Glass J. R. Paez S. (2023). Indigenous peoples and local communities as partners in the sequencing of global eukaryotic biodiversity. npj Biodivers. 2, 8–12. 10.1038/s44185-023-00013-7 PMC11062294 38693997

[B12] Mulder N. Adebamowo C. A. Adebamowo S. N. Adebayo O. Adeleye O. Alibi M. (2017). Genomic research data generation, analysis and sharing – challenges in the african setting. Data Sci. J. 16, 49. 10.5334/dsj-2017-049

[B13] Rourke M. (2017). Viruses for sale – all viruses are subject to access and benefit sharing obligations under the convention on biological diversity. Griffith University Law School Research Paper No. 17-14. 10.2139/ssrn.2984046

[B14] Sekalala S. Forman L. Hodgson T. Mulumba M. Namyalo-Ganafa H. Meier B. M. (2021). Decolonising human rights: how intellectual property laws result in unequal access to the COVID-19 vaccine. BMJ Glob. Health 6, e006169. 10.1136/bmjgh-2021-006169 PMC8277484 34253631

[B15] Sudoi A. De Vries J. Kamuya D. (2021). A scoping review of considerations and practices for benefit sharing in biobanking. BMC Med. Ethics 22, 102. 10.1186/s12910-021-00671-x 34315443 PMC8317360

[B16] Thorogood A. Rehm H. L. Goodhand P. Page A. J. H. Joly Y. Baudis M. (2021). International federation of genomic medicine databases using GA4GH standards. Cell. Genom 1, 100032. 10.1016/j.xgen.2021.100032 35128509 PMC8813094

[B17] Tone-Pah-Hote T. Redvers N. (2022). The commercialization of biospecimens from indigenous peoples: A scoping review of benefit-sharing. Front. Med. 9, 978826. 10.3389/fmed.2022.978826 PMC9386140 35991662

[B18] White M. T. (2007). A right to benefit from international research: A new approach to capacity building in less-developed countries. Acc. Res. 14, 73–92. 10.1080/08989620701290341 17844784

